# Differences in Clinical Features According to Boryoung and Karp Genotypes of *Orientia tsutsugamushi*

**DOI:** 10.1371/journal.pone.0022731

**Published:** 2011-08-15

**Authors:** Dong-Min Kim, Na Ra Yun, Ganesh Prasad Neupane, Sung Heui Shin, So Yeon Ryu, Hee Jung Yoon, Seong Heon Wie, Woo Jin Kim, Chang Youl Lee, Jong Soo Choi, Tae Young Yang

**Affiliations:** 1 Division of Infectious Diseases, Department of Internal Medicine, School of Medicine, Chosun University, Gwangju, Republic of Korea; 2 Department of Microbiology, School of Medicine, Chosun University, Gwangju, Republic of Korea; 3 Department of Preventive Medicine, School of Medicine, Chosun University, Gwangju, Republic of Korea; 4 Department of Internal Medicine, College of Medicine, Eulji University, Daejeon, Republic of Korea; 5 Department of Internal Medicine, School of Medicine, St. Vincent's Hospital, The Catholic University of Korea, Suwon, Republic of Korea; 6 Department of Internal Medicine, Kangwon National University, Chuncheon, Republic of Korea; 7 Division of Pulmonary, Allergy and Critical Care Medicine, Department of Internal Medicine, Hallym University Medical Center, Chuncheon Sacred Heart Hospital, Chuncheon, Republic of Korea; 8 Department of Internal Medicine, University of Ulsan College of Medicine, Gangneung Asan Hospital, Gangneung, Republic of Korea; 9 Department of Internal Medicine, Haenam General Hospital, Haenam, Republic of Korea; 10 Research Center for Resistant Cells, MRC, Gwangju, Republic of Korea; National Institute of Allergy and Infectious Diseases, National Institutes of Health, United States of America

## Abstract

**Background:**

Scrub typhus is an infectious disease caused by *Orientia tsutsugamushi*. The differences in virulence of *O. tsutsugamushi* prototypes in humans are still unknown. We investigated whether there are any differences in the clinical features of the Boryoung and Karp genotypes.

**Methodology/Principal Findings:**

Patients infected with *O. tsutsugamushi*, as Boryoung and Karp clusters, who had visited 6 different hospitals in southwestern Korea were prospectively compared for clinical features, complications, laboratory parameters, and treatment responses. Infected patients in the Boryoung cluster had significantly more generalized weakness, eschars, skin rashes, conjunctival injection, high albumin levels, and greater ESR and fibrinogen levels compared to the Karp cluster. The treatment response to current antibiotics was significantly slower in the Karp cluster as compared to the Boryoung cluster.

**Conclusion:**

The frequency of occurrence of eschars and rashes may depend on the genotype of *O. tsutsugamushi*.

## Introduction

Scrub typhus is an infectious disease caused by *Orientia tsutsugamushi* (*O. tsutsugamushi*) transmitted by the bites of thrombiculid mites [1]. It is characterized by abrupt fever with characteristic skin lesions as eschars. Other non-specific symptoms are headache, chills, cough, myalgia, arthralgia, and skin rashes. *O. tsutsugamushi* belongs to the family *Rickettiaceae*. It was formerly classified in the genus *Rickettsiae*, but is currently classified in the genus *Orientia*, based on structural/biological characteristics and phylogenetic analysis using ribosomal genes [2]. The four hypervariable regions of the 56-kDa protein antigen, which is located in the outer membrane of *O. tsutsugamushi*, play an important role in strain assignments [3], [4]. According to the antigenic variation, which is related to the sequence diversity of the immunodominant 56-kDa type-specific antigen, there are three prototypes of *O. tsutsugamushi*: the Gilliam, Karp, and Kato serotypes [3]. Representative strains of many serotypes, such as Kawasaki, Kuroki, Boryoung, and Shimogoshi, have also been described [4], [5], [6]. In Korea, Chang [7] isolated 137 strains of *O. tsutsugamushi*. The Boryoung serotype was predominated in the southern part of Korea, whereas the Gilliam and Karp serotypes predominated in the central part [7], [8]. By comparing the nucleotide and amino acid sequences of the 56-kDa protein variable domain, it has been shown that the Boryoung serotype, the most common serotype in Korea, has 100% homology to the Kuroki serotype and 89% homology to the Karp serotype, and that the Kuroki serotype is avirulent in mice, whereas the Boryoung serotype is highly virulent. In addition, the Kuroki serotype is strongly reactive to KP10, a Karp-specific monoclonal antibody, whereas the Boryoung serotype is non-reactive to KP10 [7], [9]. Furthermore, Groves and Osterman [10] showed that the virulence of *O. tsutsugamushi* differed depending on genetic differences between mouse strains. Nine inbred mouse strains including C3H/HeJ mice were susceptible to Gilliam infection, while six inbred mouse strains including BALB/c mice were resistant. In this mouse experiment, intraperitoneal inoculation of the Gilliam serotype resulted in a significant difference in the 50% lethal dose (MLD_50_) between the mouse strains. Nagano *et al.* [11] classified *O. tsutsugamushi* into three groups according to its virulence in mice: a highly virulent group including the Karp, Kato, and KN-3 serotypes, a low virulence group including the Kuroki, Kawasaki, and KN-2 serotypes, and an intermediate virulence group including the Gilliam serotype.

Although several studies have been conducted regarding the differences in virulence between prototypes of *O. tsutsugamushi* in mice, there have been no reports concerning differences in clinical features between the prototypes in humans. Therefore, we investigated whether there are any differences in the clinical features of scrub typhus patients infected with O. tsutsugamushi of the Boryoung and Karp genotypes.

## Materials and Methods

### Ethics Statement

The Institutional Review Board (IRB) of Chosun University, Gwangju, South Korea reviewed and approved all protocols to conduct the *Orientia tsutsugamushi* study entitled ‘Differences in Clinical Features According to Genotypes of *Orientia tsutsugamushi*’ (approval number IRB-043-27). Written, informed consent was obtained from all patients involved in this study.

### Study Design and Methods

A prospective study was conducted in patients who presented with acute febrile diseases at the Department of Internal Medicine from September to December 2006 at Chosun University Hospital (Gwangju), Haenam General Hospital (Haenam), Jangheung General Hospital (Jangheung), St. Vincent's Hospital (Suwon), Kangwon National University Hospital (Chuncheon), and Eulji University Hospital (Daejeon) in South Korea. We enrolled adult patients (aged ≥18 years) with a history of fever (>37.5°C) within the previous month and who were suspected of having scrub typhus based on either the presence of eschars or maculopapular skin rashes, or on clinical findings. The presence or absence of eschars or rashes on the patients who participated in this study was thoroughly assessed.

The diagnosis of scrub typhus was confirmed if indirect immunofluorescence antibody titers of IgM and IgG against *O. tsutsugamushi* were at least elevated four-fold during the acute and convalescent stages, or if the results of nested polymerase chain reaction (PCR) assays targeting the gene encoding the 56-kDa antigen of *O. tsutsugamushi* were positive [12].

Complications of scrub typhus were defined as follows: pneumonitis, in the presence of coughs or dyspnea together with parenchymal lung lesions or pleural effusions on chest radiographs; renal failure involving a decrease in creatinine clearance of >50% using the Cockcoft-Gault formula [13]; meningitis or meningoencephalitis with cerebrospinal fluid (CSF) counts of ≥5 leukocytes/mm^3^ together with both severe headache and neck stiffness, or an altered mental state such as confusion, obtundation, stupor, or coma with abnormal CSF cell counts without evident cause such as shock or hypoglycemia; shock with systolic arterial pressure of less than 80 mmHg or use of vasoactive drugs; GI bleeding (diagnosed by endoscopy); and death. The severity of the symptoms caused by the Boryoung and Karp genotypes was compared using the items in the modified APACHE II [14], excluding arterial pH and oxygenation. To diagnose disseminated intravascular coagulation, we used a modification of the screening tests proposed by Yu *et al.* [15] These researchers defined the diagnostic criteria for disseminated intravascular coagulation as an increase in D-dimers of ≥0.25 µg/mL or an increase in FDP of ≥10 µg/mL, whereas we defined them as an increase in D-dimers of ≥0.5 µg/mL or an increase in FDP of ≥5 µg/mL. We considered patients with increases in both D-dimers and FDP to have DIC. At presentation, a thorough history was taken, a physical examination was carried out, and hematologic laboratory tests were performed. In addition, scrub typhus-like diseases, including murine typhus, leptospirosis, hemorrhagic fever with renal syndrome, and systemic lupus erythematosus were excluded based on laboratory tests and clinical features.

### Nested PCR and DNA base sequencing

Genomic DNA for nested PCR was extracted from blood buffy coats or eschars using a QIA amp DNA mini kit (Qiagen, Hilden, Germany). Nucleotide primers were based on the nucleotide sequence of the gene encoding the 56-kDa antigen in a Gilliam serovariant of *O. tsutsugamushi*. Primers 34 (5-TCA AGC TTA TTG CTA GTG CAA TGT CTGC-3) and 55 (5-AGG GAT CCC TGC TGC TGT GCT TGC TGCG-3) were used in the first PCR, and nested PCR primers 10 (5-GAT CAA GCT TCC TCA GCC TAC TAT AAT GCC-3) and 11 (5-CTA GGG ATC CCG ACA GAT GCA CTA TTA GGC-3) were used in the second PCR amplification, generating a 483 bp fragment. Nested PCR was performed as described previously by Kim *et al* [1]. The PCR products were run on a 1.2% agarose gel. Positive samples were eluted using a QIAquick gel extraction kit (QIAGEN, Hilden, Germany) and sent to Genotech (Daejeon, Korea) for sequencing with a 3730×1 DNA analyzer (Applied Biosystems, Foster, CA, USA). The sequence of the *O. tsutsugamushi* 56-KDa gene is reported in the GenBank Database. The strains from which the amplified samples were derived were identified using BLAST of NCBI, and the sequences of the regions encoding the *O. tsutsugamushi* 56 kDA protein were analyzed using the Clustal X program. A phylogenetic tree was obtained with Tree Explorer, with bootstrap performed 1,000 times in order to increase its reliability. The Laser Gene program (DNAStar, Inc., Madison, WI) was used to compare homology between strains.

### Statistical analysis

The data collected were stored using a computer program. Continuous data are expressed as means ± SD, and the means of the two study groups were compared using an unpaired *t* test. Nominal data are expressed as frequencies or proportions, and the chi square test and Fisher's exact test were used to compare the differences in frequency between the two study groups. P values<0.05 were considered statistically significant. The statistical analyses were performed using SPSS software, version 17.0 (SPSS Inc, Chicago, IL).

## Results

### Baseline characteristics

Of the 305 patients with acute febrile diseases, 191 were confirmed as having scrub typhus by a serologic test or PCR. One-hundred sixty-eight buffy coat or eschars samples from 191 confirmed cases showed positive results by nested PCR. Of the 168 patients infected with *O. tsutsugamushi*, 133 were from Boryoung cluster and 19 from the Karp cluster. Eleven patients were infected with Kawasaki cluster, three to the Saitama cluster, and two to the Gilliam cluster. With regard to demographic characteristics, the mean age of scrub typhus patients infected with the Boryoung cluster infected patients (n = 133) was 62 years, and 38.3% of these patients were males. In the scrub typhus patients infected with Karp cluster infected patients (n = 19), the mean age was 59 years, and ten (52.6%) were males (Table 1). There were no significant differences in age, sex, and duration of illness before admission between the Karp and Boryoung clusters infected patients. Chronic diseases such as hypertension, diabetes, liver disease, and renal disease were present for more than three months in 11.3% of the Boryoung cluster and 18.2% of the Karp cluster infected patients *(p>0.05)*.

**Table 1:** Demographic data, clinical characteristics and complications of scrub typhus patients according to genotype.

Characteristics	Boryoung (n = 133)	Karp (n = 19)	*P value*
Demographic data			
Age, mean years ±SD	62±14	59±15	0.43
Male (%)	51(38.3)	10(52.6)	0.24
Duration of illness before admission, days ±SD	6±4	7±5	0.36
Chronic diseases (%)	13(11.3)	2(18.2)	0.619
Clinical symptoms and signs (%)			
Febrile sensation	130(97.7)	18(94.7)	0.42
Headache	115(86.5)	15(78.9)	0.48
Myalgia	100(75.2)	12(63.2)	0.27
General weakness	121(91.0)	11(61.1)	<0.001
Cough	49(36.8)	7(36.8)	1.00
Arthralgia	42(31.6)	3(15.8)	0.19
Chill	111(83.5)	13(68.4)	0.11
Sore throat	54(40.6)	4(21.1)	0.13
Altered mental status	12(9.0)	0(0.0)	0.36
Abdominal pain	24(18.0)	4(22.2)	0.75
Nausea/Vomiting	53(39.8)24(18.0)	3(16.7)1(5.6)	0.070.31
Fever	102(76.7)	15(78.9)	1.00
Skin rash	125(94.0)	13(68.4)	<0.001
Eschar	129(97.0)	14(73.7)	0.002
Jaundice	1(0.8)	0(0.0)	1.00
Conjunctival injection	60(45.1%)	3(15.8)	0.015
Abnormal Chest X-ray	55(42.6)	8(47.1)	0.73
Total complications (%)			
Pneumonia	31(23.3)	5(26.3)	0.78
Meningitis or meningoencephalitis	13(9.8)	1(5.3)	1.00
Shock	8(6.0)	1(5.3)	1.00
Gastrointestinal bleeding	4(3.0)	1(5.3)	0.49
Acute renal failure	23(17.3)	2(10.5)	0.74
ICU care (%)	16(12.4)	2(10.5)	1.00
Modified APACHE II score, mean±SD	7.06±3.46	6.47±3.60	0.49
Mean length of hospital stay, mean±SD	8.02±4.95	8.42±6.12	0.75
Time to disappearance of fever	25.91±19.47	56.67±43.87	0.034

Chest X-rays were not taken in 4 of the 133 patients in the Boryoung group, and two patients in the Karp group.

General myalgia, nausea/vomiting, and abdominal pain was not checked in one of the 19 patients in the Karp group.

As for clinical characteristics, the frequencies of general weakness and conjunctival injection were significantly higher in the patients infected with Boryoung cluster than in the Karp cluster (Table 1). Eschars were observed in 97% of the patients infected with the Boryoung cluster and in 73.7% of those in the Karp cluster (*p = 0.002*). The presence or absence of eschars or rashes on the patients was thoroughly assessed regardless of gender, which included the genital area, scalp, axilla, and around the breasts. Skin rashes were noted in 94% of the patients infected with the Boryoung cluster, and in 68.4% of the patients in the Karp cluster (*p<0.001*) (Table 1). There were no significant differences in patients infected with Karp and Boryoung in parameters reflecting severity including frequency of pneumonitis, meningitis or meningoencephalitis, shock, gastrointestinal bleeding, acute renal failure, the need for intensive care, and mean length of hospital stay, nor in the items of the modified APACHE II (Table 2). Although there were also no significant differences in blood cell counts, liver function tests, or urinalyses at presentation, ESR was significantly higher in the patients infected with the Boryoung cluster group than in the Karp cluster at presentation (20.64±16.9 mm/hr vs. 9.5±6.81 mm/hr, *p<0.001*). Plasma fibrinogen was significantly higher in the infected scrub typhus patients from Boryoung cluster than in the Karp cluster (328.98±86.56 mg/dL vs. 229.01±126.92 mg/dL *p = 0.002*; table 2). However, when the patients were diagnosed according to the criteria for the screening tests using D-dimers and FDP as proposed by Yu *et al.* [15], the frequency of disseminated intravascular coagulation was not significantly different in the two clusters.

**Table 2:** Routine laboratory findings in scrub typhus patients on admission.

Characteristics	Boryoung (n = 133)	Karp (n = 19)	*P value*
WBC count (no. of cells×10^3^/mm^3^)	7,976±3,869	8,210±3,236	0.80
Hemoglobin (g/dL)*	12.6±1.59	13.02±1.59	0.29
Platelet count (no. of cells×10^3^/mm^3^)	141±66.51	133±63.49	0.62
AST (IU/L)	114±107.14	165±252.40	0.40
ALT (IU/L)	96.1±97.31	147±277.22	0.44
ALP (U/L)	187.6±226.78	226.22±242.58	0.50
Bilirubin (mg/dL)	1.02±0.99	1.12±1.39	0.71
Albumin(g/dL)	36.22±375.09	3.29±0.64	0.70
LDH ( U/L)	874.7±270.45	987.86±785.56	0.60
CPK ( U/L)	259.98±659.47	143.36±132.14	0.51
ADA ( IU/L)	80.64±25.22	73.40±28.35	0.37
Serum creatinine (mg/dL)	1.23±0.58	1.27±0.97	0.78
Fibrinogen (mg/dL)	328.98±86.56	229.01±126.92	0.002
CRP (mg/dL)	9.37±7.98	8.29±6.18	0.58
ESR (mm/hr)	20.64±16.9	9.5±6.81	<0.001
PT (sec)	10.98±3.6	11.82±2.9	0.34
aPTT (sec)	30.98±11.49	32.06±9.09	0.70
DIC, no. (%) of patients	104(88.1)	8(88.9)	1.00

Values are means ±SD.

**AST** = aspartate aminotransferase; **ALT** = alanine aminotransferase; **LDH** = lactate dehydrogenase; **CPK** = creatine kinase; **ADA** = adenosine deaminase; **CRP** = C-reactive protein.

*LDH and CPK tests were performed on 130 and 125 of the 133 patients in the Boryoung group, and 14 of the 19 patients in the Karp group.

DIC tests were performed on 118 of the 133 patients in the Boryoung group, and 9 of the 19 patients in the Karp group.

Among the 19 patients infected with the Karp genotype cluster, 13 were treated with doxycycline, 3 received rifampin, one received telithromycin, and 2 were already treated with doxycycline before admission. Among the 133 infected patients with the Boryoung cluster group, 77 were treated with doxycycline, 26 received telithromycin, and 25 received rifampin. If we compare only the patients treated with doxycycline, mean fever clearance time in the patients in the Boryoung cluster was 28.9±22.67 hr in contrast to 61.27±42.86 hr (p = 0.03, 95% CI = −61.5∼−3.2) in the Karp cluster. The mean fever clearance times in all febrile patients treated with doxycycline and telithromycin or rifampin were different between the two groups (25.91±19.47 hr in Boryoung infected patients vs 56.67±43.87 hr in Karp infected, *p<.034*). We identified Karp (n = 9), Jecheon (n = 8), and Yeojoo strains (n = 2) in the Karp cluster. Consequently among the Karp cluster infected patients, presentations of fever, skin rashes, and eschars observed with the Jecheon or Yeojoo strains (n = 10) were 90, 60, and 80%, respectively, as compared to 66.7, 77.8, and 66.7% infected with the Karp strain (n = 9). Additionally, the median time to defervescence was 19 hours (range, 6–24 hours) in the patients infected with the Karp strain and 84 hours (range, 24–120 hours) in patients suffering from the Jecheon or Yeojoo strains (*P = 0.016*, Mann-Whitney test).

## Discussion

Due to limitations of serological tests for identifying *O. tsutsugamushi* serotypes, clinicians have recently preferred sequence analysis of the 56 kDa type specific antigen [6]. In this prospective study, the eschar detection rate was extremely high due to the thorough physical examination performed. Eschars and rashes observed in scrub typhus patients infected with the Boryoung cluster were 97% and 94%, respectively, in contrast to 73.7% and 68.4%, in the Karp cluster, suggesting that frequencies of eschars and rashes differ between strains. In scrub typhus patients, 5- to 20-mm eschars are formed at the sites of mite bites in the following sequence: initial maculopapules, vesicles, ulcers, and finally, eschars [16]. The presence of eschars is known to be important for diagnosis of scrub typhus [17], [18], [19]. However, the frequencies of eschars reported by investigators differ [20]. Although the eschar detection rate is relatively high in Japanese and Korean scrub typhus patients [12], [21] while it is relatively low in Thai patients having darker skin [22]. However, the difference in frequency of eschars is difficult to explain on ethnic grounds alone. It may be due to differences in research design (prospective or not), or differences between the strains of *O. tsutsugamushi* prevalent in each region. The Karp serotype is known to be present throughout Thailand, whereas the Kato serotype was found only in the southern region [23]. Ree *et al* [9] reported that the Karp strain is mainly identified in the central part of Korea and is transmitted easily in areas where *Leptothrombobidium pallidum* (*L. pallidum*) is the principal vector, whereas the Boryoung strain is mainly identified and transmitted in areas where *L. pallidum* and *L. scutellare* are found. In contrast to Ree *et al.* [9], we found the Karp strain in the southern region of Korea including Gwangju City. Chu *et al.* [24] concluded that the fatality rates of *O. tsutsugamushi* infected patients are correlated with differences in both serotypes and virulence genes. Therefore, the differences in the frequencies of eschars and rashes might be due to either the factors related to each serotype or genotype or the factors related to vectors.

In routine laboratory tests, fibrinogen levels and ESR were significantly higher in patients infected with Boryoung compared to Karp. Since it has been demonstrated that the synthesis of fibrinogen, an acute-phase reactant, increases in patients with acute inflammation [25], it seems likely that higher levels of acute phase reactants such as fibrinogen are synthesized. ESR levels were significantly higher in Boryong infected patients due to increases of acute phase reactants such as fibrinogen. Further studies on this matter are warranted.

The median time to defervescence was 25.9 hours in the Boryoung cluster infected patients in contrast to 56.7 hours in Karp cluster, and the difference was statistically significant. In Karp infected patients, the median time to defervescence was 19 hours (range, 6–24 hours) and it was 84 hours (range, 24–120 hours) in patients with the Jecheon or Yeojoo strains (*P*<0.05). Further study is needed to confirm that the Jecheon and Yeojoo strains have greater resistance to doxycycline therapy.

The main purpose of the present study was to compare the three different prototypes (Boryong, Gilliam, Karp) of *O. tsutsugamushi* prevalent on the Korean peninsula. However, only two patients were infected with Gilliam cluster in this study. Thus, we compared two main prototypes as Boryoung and Karp clusters. The nonparametric tests (demographic data, clinical characteristics, and complications) analyzed between the Boryong and Kawasaki clusters of scrub typhus infected patients were not significantly different (data not shown, *p>0.05*), thus we did not include the analysis. Groves and Osterman [10] documented that the virulence of *O. tsutsugamushi* differed significantly depending on genetic differences between mouse strains. Nine inbred mouse strains including C3H/HeJ mice were susceptible to Gilliam infection, while six inbred mouse strains including BALB/c mice were resistant to Gilliam infection. Intraperitoneal inoculation of the Gilliam genotype resulted in a significant difference in the 50% lethal dose (MLD_50_) between the mouse strains. Nagano *et al.* [11] classified *O. tsutugamushi* into three groups according to its virulence in mice: a highly virulent group including the Karp, Kato, and KN-3 genotypes, a low virulent group including the Kuroki, Kawasaki, and KN-2 genotypes, and an intermediate virulent group including the Gillian genotype. They reported that although there may be differences in the virulence of *O. tsutsugamushi* in mice and humans, deaths due to Kawasaki and Kuroki infection are rare, and most deaths in the northern part of Japan have been due to genotypes other than Kawasaki and Kuroki. They suggested that further studies on the differences in severity between different genotypes are needed to confirm their results [11], [26]. In our study, we found significant differences in frequencies of eschars, rashes, general weakness, and conjuctival injection between Boryoung and Karp cluster while no significant differences were noted in complication rates, need for intensive care, mean length of hospital stay, and severity evaluated by modified APACHE II scores. However, our study is subject to some limitations, including a low number of Karp infections. Further studies with a larger sample size are needed to confirm our findings.

In summary, this is the first study of difference between the clinical features of scrub typhus patients infected with the Boryoung and Karp genotypes. We noted that eschars and rashes were found in 97% and 94% of the patients infected with the Boryoung cluster, respectively, in contrast to 73.7% and 68.4% of the patients in the Karp cluster, respectively, suggesting that the frequency of eschars and rashes in scrub typhus patients may depend on the genotypes of *O. tsutsugamuchi*.
